# Morphological and molecular evidence for functional organization along the rostrocaudal axis of the adult zebrafish intestine

**DOI:** 10.1186/1471-2164-11-392

**Published:** 2010-06-22

**Authors:** Zhengyuan Wang, Jianguo Du, Siew Hong Lam, Sinnakarupan Mathavan, Paul Matsudaira, Zhiyuan Gong

**Affiliations:** 1 Computation and Systems Biology, Singapore-MIT Alliance, 117543, Singapore; 2 Department of Biological Sciences, National University of Singapore, 117543, Singapore; 3 NUS Centre for BioImaging Sciences, National University of Singapore, 117543, Singapore; 4 Dept of Oceanography, Xiamen University, Xiamen, 361005, China; 5 Third Institute of Oceanography, State Oceanic Administration, Xiamen, 361005, China; 6 Genome Institute of Singapore, 60 Biopolis Street, 138672, Singapore

## Abstract

**Background:**

The zebrafish intestine is a simple tapered tube that is folded into three sections. However, whether the intestine is functionally similar along its length remains unknown. Thus, a systematic structural and functional characterization of the zebrafish intestine is desirable for future studies of the digestive tract and the intestinal biology and development.

**Results:**

To characterize the structure and function of the adult zebrafish intestine, we divided the intestine into seven roughly equal-length segments, S1-S7, and systematically examined the morphology of the mucosal lining, histology of the epithelium, and molecular signatures from transcriptome analysis. Prominent morphological features are circumferentially-oriented villar ridges in segments S1-S6 and the absence of crypts. Molecular characterization of the transcriptome from each segment shows that segments S1-S5 are very similar while S6 and S7 unique. Gene ontology analyses reveal that S1-S5 express genes whose functions involve metabolism of carbohydrates, transport of lipids and energy generation, while the last two segments display relatively limited function. Based on comparative Gene Set Enrichment Analysis, the first five segments share strong similarity with human and mouse small intestine while S6 shows similarity with human cecum and rectum, and S7 with human rectum. The intestinal tract does not display the anatomical, morphological, and molecular signatures of a stomach and thus we conclude that this organ is absent from the zebrafish digestive system.

**Conclusions:**

Our genome-wide gene expression data indicate that, despite the lack of crypts, the rostral, mid, and caudal portions of the zebrafish intestine have distinct functions analogous to the mammalian small and large intestine, respectively. Organization of ridge structures represents a unique feature of zebrafish intestine, though they produce similar cross sections to mammalian intestines. Evolutionary lack of stomach, crypts, Paneth cells and submucosal glands has shaped the zebrafish intestine into a simpler but unique organ in vertebrate intestinal biology.

## Background

The surface of the intestine epithelium is the site where nutrients are absorbed into the body. This absorption function is aided by expanding the surface area of the gut into villi at the tissue level and microvilli at the cellular level. Consequently, the mouse and human intestine has become a model for studying how this large surface develops during embryogenesis, the role of stem cells in the renewal of the epithelium, and development of colorectal cancer [1-3]. However, these complex problems can be studied in a simpler system, the zebrafish (*Danio rerio*), which has emerged as an important vertebrate model for study of not only human development but also diseases [4-6].

So far, morphological development of zebrafish intestine has been relatively well characterized in embryos and larvae [7-11], However, the organization and physiology of digestive tract has not been specifically documented for adult zebrafish although several books are available for description of general fish intestine anatomy [12-14]. Zebrafish, like many fish, lacks a morphologically and functionally distinct stomach and does not express genes that encode specific gastric functions [9-12]. Sections of intact zebrafish embryos and juveniles from whole animal serial sections and microCT tomography reveal the digestive tract from pharynx and esophagus to the three sections of the folded intestine and anus [15-17]. Previous studies have described the zebrafish intestine as a tapered tube that begins at the esophageal junction and is folded into three sections, the large diameter rostral intestinal bulb, the mid-intestine, and the small diameter caudal intestine [9]. However, it is not known whether these regions are functionally distinct or whether their functions correspond to the mammalian stomach, small intestine or large intestine. In this study, we characterized the anterior-posterior axis of adult zebrafish intestine at tissue, cellular and molecular levels. By comparing the morphological and molecular characteristics, we identified structurally and functionally distinct areas that correspond to the small intestine and large intestine but not stomach.

## Results

### Architectural differences along the zebrafish intestinal tract

**Figure 1:**
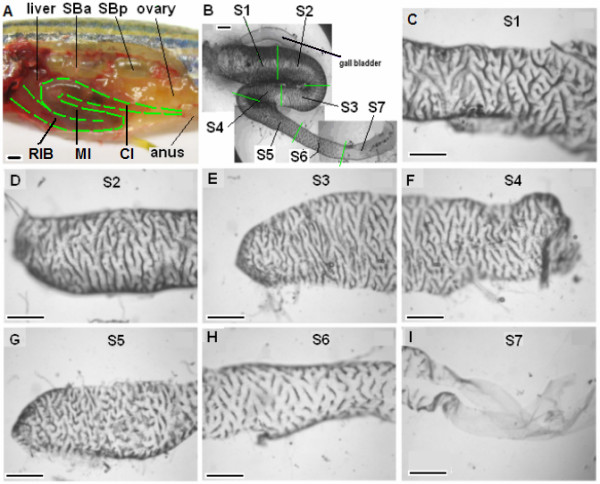
**Anatomical features of adult zebrafish intestine**. (A) A partially dissected 6-month-old zebrafish to show the folding of the three portion of intestine in vivo: rostral intestinal bulb (RIB), mid-intestine (MI) and caudal intestine (CI). Liver, ovary, anus, swimbladder anterior (SBa) and posterior (SBp) chambers are indicated. (B) An isolated zebrafish intestine in vitro after removal of the surrounding mesentery. The isolated intestine was divided into seven roughly equal-length segments as indicated by green lines: S1-S2 from RIB, S3-S4 from MI and S5-S7 from CI. The associated gall bladder is indicated. (C - I) Surface views of segments S1-S7 showing the folding of the mucosal surface into circumferential ridges. Scale bars, 500 *μ*m.

The anterior-posterior axis of the digestive tract has been previously described for embryonic and juvenile zebrafish [7] and cyprinids [15,16]. In summary, the adult digestive tract consists of the mouth, pharynx, esophagus, intestine, and anus (Additional file 1). However, the zebrafish belongs to the group of stomach-less fishes in which the intestine transits directly from esophagus. In adult fish, it is folded into three sections: the rostral intestinal bulb, mid-intestine and caudal intestine (Figure 1A), as previously reported by Wallance et al [9]. When dissected from the animal and freed of the surrounding mesentery, the intestine remains folded by two turns into three straight regions that correspond anatomically to the three portions as observed in vivo (Figure 1B). Their diameters decrease along the anterior-posterior axis (Figure 1B).

To characterize intestinal function, we subdivided the intestinal bulb, mid-intestine, and caudal sections into seven segments, S1-S7 (Figure 1B) and examined their architecture under a light microscope. We observe that the zebrafish intestine surface in segments S1-S6 is covered by ridges that are oriented circumferentially across the intestine axis (Figure 1C-1I, as previously reported [9]. The ridges are densely packed and highly branched. In segment S6 the ridges are shorter and broader than the anterior segments. Segment S7 is morphologically distinguished from the other six segments by a smooth surface devoid of any folds or villus-type structures (Figure 1I).

**Figure 2:**
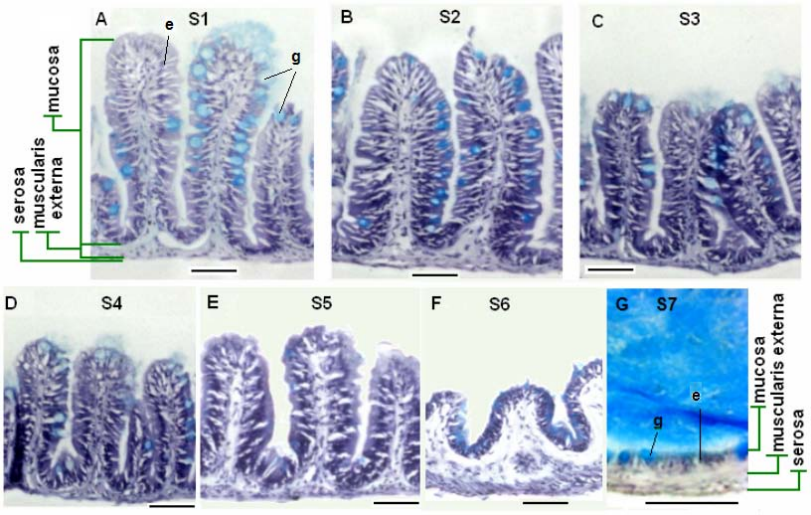
**Histological features of adult zebrafish intestine along the seven anterior-posterior segments**. (A-G) Representative cross sections of intestine from segments S1-S7 respectively. All sections were stained by Hematoxylin/Eosin/alcian blue. Segments S1-S6 contain three tissue layers: mucosa, muscularis externa and serosa, while S7 has a simple epithelium directly adjacent to the muscularis externa. Goblet cells (stained blue) are interspersed among the absorptive cells. Examples of enterocytes (e) and goblet cells (g) are indicated in Panels (A) and (G). Scale bars: 50 *μ*m.

Cross-sections of the intestinal segments reveal a simple architecture for the zebrafish digestive tract of a mucosa, muscularis externa and serosa layer (Figure 2). The intestinal mucosa consists obviously of columnar-shaped enterocytes and mucous-secreting goblet cells. Other types of cells such as enteroendocrine cells may be identified by special staining or GFP transgenic labelling [8-10]. An underlying lamina propria contains blood capillaries, lymphatic vessels, muscle fibres and mesenchymal cells. The general architecture of zebrfish intestine, as revealed through cross-sections, resembles that of the mammalian intestine as described previously [9]. The mucosal layer is directly ensheathed by circular and longitudinal smooth muscle tiers of the muscularis externa within which are embedded the plexus of myenteric neurons as reported previously [8,9]. In the mammalian duodenum, a typical submucosal layer contains Brunner's glands, the branched tubular or branched tubuloalveolar glands that produce alkaline secretions to neutralize the acidic chime entering the duodenum [17]. However, in the zebrafish intestine the submucosa layer and Brunner's glands are absent (Figure 2).

Consistent with our earlier observations of intestinal ridges in segments S1 to S6 and absence of ridges in S7, these ridges in cross-section resemble the spatially separate villi in the mouse or human small intestine (Figure 2). In contrast to the mammalian intestine, crypts are absent from the base of the ridges and specialized crypt cells such as Paneth cells are not observed [9]. The villar ridges are comparable in height from segments S1 to S5 (Figure 2, shorten and broaden in segment S6 (Figure 2F), and absent in segment S7 (Figure 2G). Segments S5-S7 often contain compact excretions that are ensheathed by a mucous layer (stained blue by Alcian Blue in Figure 2G). In addition to the absence of villi, segment S7 is distinguished by its lining of abundant goblet cells that are interspersed by absorptive epithelial cells (Figure 2G). The muscularis externa is apparent, but the mucosa layer, in general, appears very thin compared with other segments of the intestine. Thus, based on histology and architecture, the intestinal lining is divided into three morphologically distinct regions, segments S1-5, S6, and S7.

### Distinct molecular signatures along the zebrafish intestinal tract

**Figure 3:**
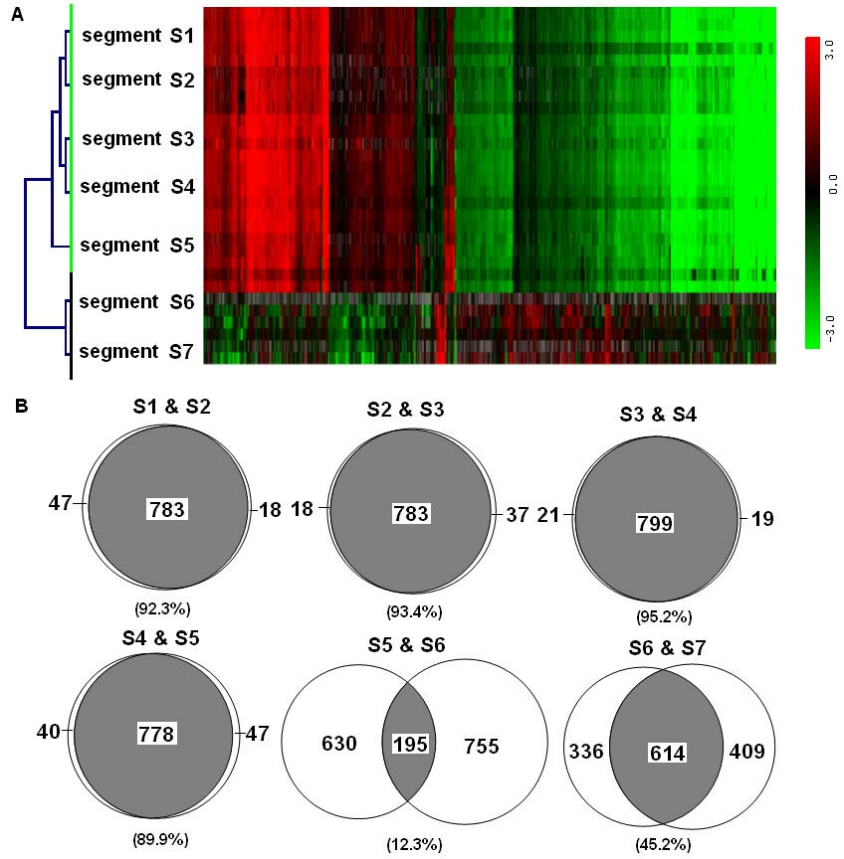
**Anayses of genes differentially expressed along the anterior-posterior intestine**. (A) Hierarchical clustering of segments S1-S7 by differentially expressed genes selected by ANOVA analysis. Segments S1 to S5 are clustered as one group; segments S6 and S7 are clustered as another group. (B) Overlap analysis of up-regulated genes in adjacent segments. The number and percentage of overlapping genes are indicated within and below the intersection respectively.

Based on gross morphology, segments S1-S5 are similar while segments S6 and S7 are different. These differences in structure suggest that there should be inherent differences in function. To test this idea, we examined and compared the molecular signatures of each segment by profiling their transcriptional activity. Using a standard Bonferroni corrected p-value < 0.1 (adjusted for false discovery) applied to results from a one-way ANOVA analysis, we identified 2,558 genes that were differentially expressed in at least one of the seven segments and organized the genes by hierarchical clustering analysis [18] (Figure 3A) for similarities in patterns of gene expression. This analysis sorted the seven segments in their anatomical sequence, S1-S7 with S1-S5 more similar to each other than to segments, S6 and S7.

To understand the significance of the clusters, we then applied a threshold of 2.0 fold against pooled RNA extracted from whole adult zebrafish to the set of 2,558 genes from the ANOVA analysis. This analysis shows the numbers of genes that are abundantly expressed in each individual segment are: 830 (S1), 801 (S2), 820 (S3), 818 (S4), 825 (S5), 950 (S6) and 1023 (S7). To determine the extent to which genes are commonly expressed along the intestinal tract, we determined the overlap in gene sets between pairs of adjacent segments (Figure 3B). Consistent with the clustering results in Figure 3A, significant intersection was found between segments S1-S5 [more than 700 genes (or ≥89.9%) for each overlap, Figure 3B]. However, segments S6 and S7 express quite different sets of genes than the anterior segments. S5 and S6 overlap in 12.3% genes while S6 and S7 share only 45.2% genes in common. Similar results were also observed from analysis of down-regulated genes (Additional file 2).

**Figure 4:**
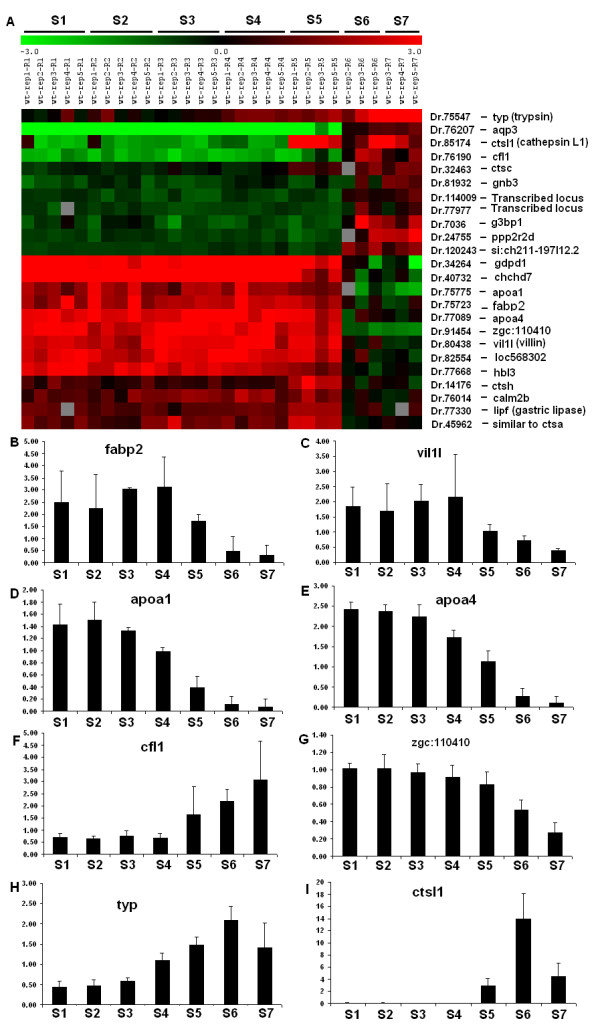
**Expression patterns of selected intestinal genes**. (A) Expression patterns of selected genes based on microarray data. The genes were selected based on their known function in the digestive tract and/or from their expression profiles. (B-I) qRT-PCR validated expression pattern of selected genes. The histograms show the relative changes of the gene expression levels compared with their respective levels of the housekeeping gene *bactin2*. Gene names are indicated in each panel.

To confirm these patterns of overlap, we identified a number of genes that were either highly expressed in segments S1-S5 (e.g. *gdpd1, chchd7*, zgc:11410, *hbl3*, etc) or in segments S6 and S7 (e.g. *trp*, *ctsl1, ctsc, gnb3, gsbp1, ppp2r2 d*, etc), suggesting a comprehensive functional transition along the zebrafish intestine (Figure 4A). The expression patterns of *vil1l *(Figure 4B), *fabp2 *(Figure 4C), *apoa1 *(Figure 4D), *apoa4 *(Figure 4E), *cfl1 *(Figure 4F), zgc:110410 (Figure 4G), *typ *(Figure 4H) and *ctsl1 *(Figure 4I) were confirmed by real time RT-PCR. Thus, based on the molecular analyses, the zebrafish intestine can be divided into three molecularly distinct regions as represented by segments S1-S5, S6, S7, respectively.

### Molecular features of the small and large intestine-like functions

Having shown the intestine can be subdivided into three regions, S1-S5, S6, and S7, based on similarities in expressed genes, we investigated whether the identity of the genes gives insight into intestine function and thus we selected several intestinal marker genes for more detailed analysis.

The functions of the mouse and human small intestine have been characterized by well known molecular markers including *fabp2 *[19], *vil1l *[20-22], *apoa1 *and *apoa4 *[23-25]. All of these genes were detected in S1-S5 in our microarray analyses by their higher levels of expression compared with total RNAs from whole fish (Figure 4A) and confirmed by real time RT-PCR analyses (Figure 4B-E). Intestinal *fabp2 *gene encodes a fatty acid binding protein that is specifically involved in the intracellular transport of fatty acids in the small intestine [19,26,27]. This gene is highly conserved in teleosts, amphibians, avians and mammals [28]. Previously, an *RFP *transgenic zebrafish line under the intestinal *fabp2 *promoter, *Tg(fabp2:RFP)*, has been generated and RFP reporter gene is specifically expressed in the intestine [29]. To further verify the expression of intestinal *fabp2*, we isolated an intestine from a 3-month-old *Tg(fabp2:RFP) *transgenic zebrafish and found that RFP fluorescence was high in segments S1-S4, but quickly diminished around the second turn of the intestine (Additional file 3, Panel B). This expression pattern was also confirmed by direct detection of endogenous *fabp2 *mRNA expression by *in situ *hybridization (Additional file 3, Panel C). Closely resembling the pattern of *fabp2 *expression, villin expression is also restricted to the mammalian gastrointestinal tracts where it is highly expressed in the small intestine [21]. Our microarray data show that zebrafish villin gene (*vil1l*) is highly expressed in segments S1-S5 and its expression is reduced in segments S6 and S7. This finding is further validated by real time RT-PCR (Figure 4C), where the expression of *vil1l *decreases in segment S5 and to a negligible level in segments S6 and S7. In further support that segments S1-S4 possess features of a small intestine, another two conserved markers, *apoa1 *and *apoa4*, also showed similar expression pattern to *fabp2 *and *vil1l *genes along the anterior-posterior axis of zebrafish intestine. These two genes can also be considered to be reliable molecular markers for small intestine because in 36 human tissues and 45 mouse tissues examined, expression of mammalian *Apoa1 *and *Apoa4 *are highly restricted to the digestive organs including small intestine and liver (GSE2361 and GDS182, GEO database, NCBI). These patterns of small intestine markers together with the transcriptome data suggest that the small intestine comprises segments S1-S4 and transition into a different function occurs in segment S5.

If segments S1-S4 are small intestine-like, then we investigated whether S5-S7 expresses gene markers for the large intestine. Two genes, *cfl1 *(*cofilin1*) and *aqp3 *(*aquaporin 3*), distinguish segments S5-S7 from S1-S4. *Cfl1 *belongs to a family of actin-binding proteins and mediates dynamic stabilization of actin filaments [30]. Our microarray and real time RT-PCR data (Figure 4A, F) indicate that *cfl1 *is primarily expressed in segments S5-S7, but down-regulated in the first four segments. Analysis of rat EST database suggests that *cfl1 *is expressed in the large intestine but not in the small intestine (Unigene's EST profile viewer, Unigene Rn.11675, NCBI). Similarly, our microarray data indicated increased expression of Aqp3, an osomoregulatory channel protein on membrane of epithelial cells [31], particularly in the large intestine of mammals [32] and mucus cells of the posterior intestine of teleost eel [33], in segments S6 and S7 (Figure 4A, Dr.76207). Aquaporins are water channel proteins that facilitate water movement, hence increase water permeability, across cell membrane and the increase expression of Aqp3 is therefore important for absorption of water in the intestine, in particular, for the faecal dehydration in the large intestine. In line with this, the mammalian aquaporin 3 is expressed along the gastrointestinal tract, with its highest expression in the colon [31,32]. Thus, while segments S1-S5 possess molecular features of small intestine, segments S6 and S7 have molecular features of large intestine with segment S5 as a transitional region).

### Analysis of gene ontology (GO) along the anterior-posterior axis

To infer the functions of the intestinal segments, 2-fold up-regulated genes against the whole fish reference RNA were extracted for the three groups, S1-S5 (891 genes), S6 (1147 genes), and S7 (1107 genes). We envision that these significantly up-regulated genes should better represent specific functions of different intestinal segments and thus gene ontology analyses were carried out for these genes using GOTree Machine [34]. Significantly enriched (p-value < 0.01) categories in each region are shown in Additional file 4.

As expected from a major metabolic organ, the intestine of zebrafish harbours a rich collection of genes involved in metabolism, molecular transport and localization, catalytic activities among others. However, major differences were found between the three groups of S1-S5, S6 and S7. There are 56 categories that were statistically enriched in S1-S5, but only 6 categories in S6 and 8 categories in S7. Among these enriched categories, up-regulated genes in S1-S5 are involved in a wide range of metabolic processes, including metabolism of fatty acid, organic acid, lipid, vitamin, heme, alcohol, glucose, hexose, monosacchride, carbohydrate, etc. (Additional file 4). They also play important roles in energy generation and homeostasis of ion, iron and cations. Notably, a group of genes are associated with catalytic activities such as hydrolase activity and transferase activity, which are important for the absorptive function of the small intestine. The variety of GO categories in the S1-S5 group supports the multiple functions of this part of zebrafish intestine with features of the small intestine (to be discussed below).

The S6 and S7 groups, on the other hand, only show a few statistically enriched categories. For example, genes from S6 are involved in oxidoreductase activities while genes from S7 are enriched in biosynthesis of vitamin and pyridine nucleotide (Additional file 4). They are also involved in intracellular signaling and pentosyl/phosphoribosyl transferase activity. It seems that segments S6 and S7 represent two regions of zebrafish intestine that perform tasks apparently different from those of S1-S5.

### Cross-species Gene Set Enrichment Analysis (GSEA) indicates the segments S1-S5 to be multi-functional

An independent approach to confirm the identity of the intestine sections as small and large intestine is by taking the three gene set pools from S1-S5, S6, and S7 and comparing them by GSEA analysis against the whole transcriptomes of the mouse and human stomach, small intestine and large intestine (GDS182 and GSE2361, GEO database, NCBI). Results, summarized in Table 1, show segments S1-S5 closely resemble the small intestines of mouse and human with highly significant FDR values (<0.001). Segments S1-S5 show little resemblance to stomach (mouse FDR = 0.06; human FDR = 0.68) and no resemblance to the human cecum. Gene ontology analysis shows that majority of the genes corresponding to the leading edge of the GSEA curve are involved in the metabolism of lipid, fatty acid, cholesterol and glycerolipid, or involved in peptidase, oxidoreductase activity, reminiscent of the activities of the mammalian small intestine (data not shown).

**Table 1:** Comparison of transcriptome similarity of zebrafish intestinal segments and human/mouse intestines by GSEA analyses

Human/mouse intestines	**Zebrafish intestinal segments**^**#**^			GEO accession
		
	S1-S5	S6	S7	
Mouse stomach	0.06*	1.00	0.94	GDS182

Human stomach	0.68	0.63	0.55	GSE2361

Mouse small int.	<0.001***	0.34	0.40	GDS182

Human small int.	<0.001***	0.21*	0.86	GSE2361

Mouse cecum	0.17*	0.28*	0.21*	GDS182

Human cecum	1.0	<0.001***	0.016**	GSE9254

Human colon	<0.001***	1.00	0.90	GSE2361

Human sigmoid colon	<0.001***	0.015**	0.038**	GSE9254

Human rectum	<0.001***	<0.001***	0.003***	GSE9254

^#^Numbers represent False Discovery Rate (FDR): *** highly significant (p < 0.01); ** significant (p < 0.05); * marginally significant (p < 0.25).

In contrast to segments S1-S5, segment S6 closely resembles the cecum and rectum of the human large intestine (FDR < 0.001), while segment S7 resembles human rectum only (FDR = 0.003). Gene ontology analysis shows that S6 resembles human cecum in glycolysis, oxidoreductase activity, metabolism of amino acid, amine derivative, organic acid, carboxylic acid and alcohol. While in S7, metabolism of membrane lipid was found to be enriched. Water retention is a common function in mammalian large intestine. Consistent with this, several aquaporin genes, including *aquaporins 1, 3 *and *10 *are highly expressed in S6/S7. In particular, Aquaporin 3 is well known a key component of faecal dehydration in mammalian colon [31,32]. Interestingly, although S1-S5 most closely resembles the small intestine, we detected some significant similarity with functions found in the human colon and rectum (Table 1), suggesting that the zebrafish segments S1-S5 may have broader functions. This phenomena may reflect the less specialized and differentiated features of fish intestine as a primitive species of vertebrates. This is particularly true for fish "small intestine" (S1-S5) as it constitutes more than two thirds of the length of the intestine.

In summary, GSEA analysis supports that segments S1-S5 of zebrafish intestine possess features of a mammalian small intestine, while segments S6 and S7 possess features of a mammalian large intestine (with S7 resembling rectum in particular).

### Stomach-like functions of the intestine

**Figure 5:**
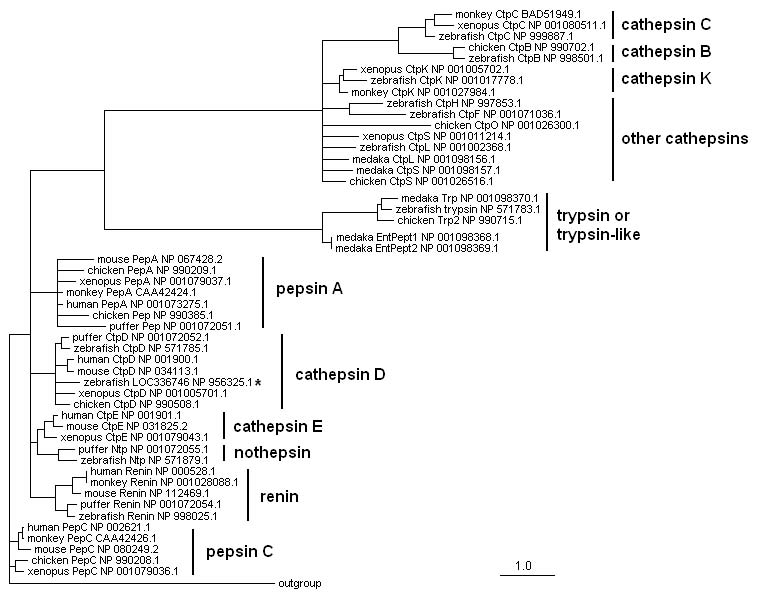
**Phylogenetical analysis of zebrafish genes encoding aspartic proteases**. The amino acid sequences of zebrafish digestive proteases were compared with those from other species, including mammals, amphibians and fishes. The parasite aspartic protease (*Haemonchus contortus*), CAA96571, is used as the outgroup. * indicates a candidate hypothetical protein product.

A striking feature of the zebrafish anatomy is the absence of stomach [9,35]. To understand whether the gut carries out a cryptic gastric function, we examined the zebrafish genes encoding enzymes including pepsin and some digestive proteases with implications of functions of a stomach. Mammalian pepsinogens are classified into three major groups and two minor groups [36,37], however, a pepsinogen gene in zebrafish has never been reported. We searched for potential pepsinogen sequences in the zebrafish genome. First, we conducted a BLAST search against the Ensembl genome database http://www.ensembl.org using sequences of human PGC (PEPSINOGEN C) and PGA (PEPSINOGEN A) but did not detect any significant hits relevant to the pepsinogen gene. Then in a more specific TBLASTN search [38] using the pfam00026 domain that is well conserved across all aspartic proteases in vertebrates, we identified pepsinogen genes as well as some aspartic protease genes in human, mouse, Xenopus and Fugu fish, together with a few zebrafish aspartic protease genes and a putative gene sequence encoding a hypothetical protein NP956325.1. To determine whether these zebrafish sequences could represent a pepsinogen gene, relevant amino acid sequences were aligned, a phylogenetic tree was constructed using quartet puzzling algorithm implemented in the Tree-Puzzle program [39], and the result was visualized by TreeViewX [40]. Our analysis suggests that the zebrafish has genes coding for rennin, nothepsin and several members of cathepsins (Figure 5). However, none of these zebrafish genes resemble the mammalian pepsinogen genes. The genome search results and phylogeny analysis results together suggest that the pepsinogen gene locus is not present in the zebrafish genome.

Whereas pepsinogen is not encoded in the zebrafish genome, other stomach markers may be expressed by the intestine. For example, *lipf *is a gastric lipase gene encoding an acidophilic lipase known to be secreted by mammalian gastric chief cells [41,42]. Its expression in human is restricted to esophagus, stomach and several other tissues, but not in the intestine (Unigene's EST profile viewer, UniGene Hs.523130, NCBI database). In contrast, *lipf *is expressed in all seven segments of the zebrafish intestine and not restricted to any particular segment (Figure 4A).

## Discussion

For comparison of similarity between zebrafish fragments, we used differentially expressed genes generated by ANOVA (Figure 2 and Additional file 2). For GO (Additional file 4) and GSEA (Table 1) analyses, we selected 2-fold up-regulated genes against the reference RNA (total whole adult fish RNA) and this selection was independent of the initial set of differentially expressed genes selected by ANOVA for similarity analyses. Our approach would filter out ubiquitously expressed housekeeping genes. Abundantly expressed intestine-specific or enriched transcripts will be retained by the 2-fold selection as their concentrations in the total fish RNA pool would be diluted much more than 2 fold. We have also tried 1.5-fold selection, basically the same GSEA results were obtained (data not shown).

The results in this study document that the zebrafish intestine is regionally segmented into a small intestine and large intestine. This conclusion is supported by morphology and three lines of independent analysis of gene expression profiles from seven segments of the intestine. Clustering analysis reveals a general similarity between S1-S5 and differences between S6 and S7 and the degree of similarity is measured by the degree of overlap in gene sets expressed in neighboring segments. Second, we showed that well-known markers of the mammalian small and large intestine such as *villin, fabp2*, and *cof1 *are differentially expressed along the anterior-posterior axis. Finally by ontologies of genes expressed in the segments are consistent with small and large intestine function and confirmed by whole transcriptome comparisons with human and mouse small and large intestine gene sets. Based on these findings, we suggest that the intestinal bulb, mid-intestine, and the anterior third of the caudal intestine corresponds to the small intestine of the mammalian gut while the remaining posterior portion of the caudal intestine corresponds to the large intestine terminating with the rectum.

In comparison with the mammalian intestine, the zebrafish intestine has a simple architecture with the intestinal lining folded into villar ridges rather than distinct finger-shaped villi of the mammalian small intestine. In cross section, a ridge appears identical to a villus and thus may be an evolutionary precursor to discrete villi. In support of this idea, an intermediate stage (from D8-D8.5) in the morphogenesis of the chick intestine includes the initial formation of longitudinally oriented previllous ridges that buckle into a zig-zag pattern and eventually form villi in adult intestine. Thus, in birds, ridges are embryological precursors to villi [43].

In addition to the lack of well-defined villi, the zebrafish intestine lacks well-defined crypts, infoldings of the intestinal surface where stem cells and proliferating cells are located [8,9]. Intestinal crypts are normally found at the base of the villus or lining the colon of the large intestine. In the zebrafish intestine, mitosis is restricted to the base of the villar ridges ([9,10] and our unpublished data), suggesting that the Crypts of Lieberkuhn are specializations of the mammalian intestine. This arrangement also raises questions about the dynamics of epithelial renewal because cell proliferation in the intestine is balanced by apoptosis at the villus tips. Location of apoptosis in the adult zebrafish intestine is rarely reported but we find most cell death occurs in the distal portion of the villar ridges and apoptosis is much more active when compared with mammalian intestines (our unpublished data). In contrast, apoptosis in the embryonic and larval zebrafish intestine goes undetectable until morphogenesis has completed [8,9], while apoptosis occurs throughout the development of the mouse duodenum [44] but is reduced to a few cells per villus during adulthood [45].

Like many other fish including cyprinids and others [46], the zebrafish has evolved into a stomachless fish [9]. The absence of a functional pepsinogen gene from the digestive tract is not unique to zebrafish but also occurrs in medaka fish (*Oryzias latipes*) and other stomach-less fishes. Interestingly, the expression of pepsinogen gene in another stomchless fish, the puffer fish (*Takifugu rubipes*), is restricted to its skin tissue, adopting different functions [35]. Without a stomach, digestion and absorption must begin as early as possible in the limited length of the zebrafish digestive tract. Ingested food is temporarily stored in the rostral intestinal bulb that bulges like an elastic sac, where food starts to be broken down in the absence of a stomach [47] and the entire length of the intestine may serve to degrade food.

Based on analysis in larval zebrafish and other cyprinids, previous reports raised that the posterior zebrafish intestine may be analogous to the mammalian colon [48,49]. This has also been proposed in a recent study based on histological data and molecular markers [9]. Here our transcriptome data provide more solid evidence that this part of intestine in adult zebrafish resembles mammalian colon and rectum and moreover, segments S6 and S7 distinguish themselves from each other.

## Conclusions

In the present study, the entire intestine of adult zebrafish was systematically examined at the levels of anatomy, histology and transcriptome. Despite the lack of crypts and evident structural distinction throughout most of the length of intestine, our genome-wide gene expression data have shown that the rostral, mid, and caudal portions of the zebrafish intestine have distinct functions analogous to the mammalian small and large intestine, respectively. Organization of ridge structures represents a unique feature of zebrafish intestine, though they produce similar cross sections to mammalian intestines. Evolutionary lack of stomach, crypts, Paneth cells and submucosal glands has shaped the zebrafish intestine into a simpler but unique organ in vertebrate intestinal biology. This scenario may represent an evolutionary primitive feature of the digestive tract, where functional regionalization precedes morphological regionalization in a low vertebrate.

## Methods

### Maintenance of zebrafish and dissection of zebrafish intestine

*Danio rerio *of about one year old were maintained following established protocols [50] and in compliance with Institutional Animal Care and Use Committee (IACUC) guidelines. Zebrafish were euthanized by 0.1% 2-phnoxyethanol and their intestines were isolated and cut into seven segments along the anterior and posterior axis, as shown in Figure 1B. The seven segments were labeled S1, S2, S3, S4, S5, S6 and S7, respectively.

### Paraffin sectioning of zebrafish intestine

Intestinal segments were fixed in 4% paraformaldehyde in phosphate buffered saline at room temperature overnight. Then the intestine samples were dehydrated in 70% ethanol overnight and further dehydrated in ethanol with increasing gradients (75%, 90%, 95% and 100%). The samples were cleared in 100% Histoclear II (National Diagnostics, US) for 30 min twice, embedded in liquid paraffin at 58°C for 30 min, then changed into fresh paraffin for final embedding at 58°C overnight. Finally, the samples were sectioned at 7 *μ*m on a Reichert-Jung 2030 microtome (Leica, Germany) and collected onto Fisher SuperFrost slides. The slides were left on a heating block at 42°C overnight before further assays were conducted.

### Hematoxylin & Eosin & Alcian blue staining

Tissue sections were stained by Meyer's hematoxylin for 10 min, rinsed in tap water, stained by eosin for 1 min, followed by dips in acidic ethanol and rinse in tap water. They were stained by alcian blue (Biogenex, US) for 10 min and rinsed in tap water. Finally, the slides were dehydrated in ethanol of increasing gradients (75%, 90%, 95% and 100%), cleared by HistoClear II (National Diagnostics, US), mounted with DePeX (EMS, US) mounting medium and covered by coverslips. Images were taken using a Zeiss Axiovert imaging system.

### Quantitative real-time PCR

Quantitative real-time PCR was carried out in 96-well plates on a LightCycler 480 system (Roche, Swiss). The PCR reaction was set up according to the manufacturer's protocol with optimization of primer-specific annealing temperature and extension time. PCR products were labeled by SYBR Green dye. All gene expression levels were measured and normalized against the level of house-keeping gene *bactin1 *expression.

### Microarray experiments

Intestines were isolated from male adult zebrafish, quickly rinsed in 1× phosphate buffered saline/diethylpyrocarbonate, and cut into seven segments according to Figure 1B. To maintain a more homogenous molecular background, only male fish were used for microarray analyses. The same segments from every 10 fish were pooled as one biological replicate and they were kept in liquid nitrogen till extraction of RNA. RNA was double-extracted using Trizol (Invitrogen, USA). A total of five replicates were prepared for each segment. For each replicate, 10 *μ*g RNA was reverse-transcribed into cDNA with incorporation of aminoallyl-dUTPs. Later the samples were hybridized onto in-house spotted microarray chips with labeling by Cy5 as described previously [51]. RNAs from whole fish were used as reference for all experiments and labelled by Cy3. The arrays contained 22 K oligonucleotide probes where 16.4 K probes were designed by Compugen (USA) [51] and the remaining probes were in-house designed by the bioinformatics group in Genome Institute of Singapore (GIS). The complete list of gene probes has been submitted to GEO database with the access number GSE20884. For each gene probe, one 65-mer oligonucleotide probe was designed from the 3' untranslated region for maximizing gene specificity. 172 copies of the beta-actin gene probe were included in each array as calibration spots. All of the oligonucleotide probes were synthesized from Sigma and spotted on glass slides using the Genemachine robotic spotter/arrayer (Genomic Solutions, Ann Arbor, MI, USA) housed at GIS [52]. After hybridization, the microarray chips were scanned and graded, and raw data went through two rounds of normalization: They were first normalized within each single array and second normalized across the whole collection of arrays, using LOWESS method implemented in Gene Cluster 3.0 during the pre-processing stage.

### Identification of differentially expressed genes from the microarray data

Pre-processed microarray data were visualized and subjected to one-way ANOVA test using MeV MultiExperiment Viewer software [18]. One-way ANOVA test was performed using a critical p-value of 0.1 with standard Bonferroni correction for all seven intestinal segments. The selected genes were used for clustering and expression pattern analysis to compare the similarity and differences in the seven segments.

### Gene ontology (GO) analysis by GO Tree Machine

Up-regulated genes were selected for each individual segment based on the fold changes of their expression levels (at least two told up against the RNA of pooled adult zebrafish and FDR adjusted p-value < 0.05). Gene ontology was carried out using GOTree Machine, which is a web-based tool developed by the Vanderbilt University to analyze gene ontology for a given set of genes [34]. It compares the distribution of genes in the gene set of interest in each GO category to those in the reference gene set, i.e. the transcriptome of zebrafish in our case. Gene information was retrieved from GeneKeyDB, a database that integrates gene information from Ensembl, Swiss-Prot, HomoloGene, Unigene, Gene Ontology Consortium and Affymetrix etc. Statistical tests were used for the assessment of enrichment of each gene category.

### Gene Set Enrichment Analysis (GSEA)

GSEA is a computational method that determines whether a priori defined set of genes shows statistically significant, concordant differences between two biological samples; it calculates an enrichment score using a running-sum statistic through a ranked list of gene expression data set [53]. In this work, the software GSEA2.0 developed by the Broad Institute [53] was used. The statistical significance of the enrichment score was estimated by using an empirical phenotype-based permutation test procedure. A false discovery rate was provided by introducing adjustment of multiple hypothesis testing.

## Authors' contributions

Conceived and designed the experiments, and analyzed the data: ZW JD PM ZG. Contributed reagents/materials/analysis tools: SHL SM. Wrote the paper: ZW PM ZG. All authors read and approved the final manuscript.

## Supplementary Material

Additional file 1 **Anatomy of adult zebrafish showing the digestive tract**. A composite of H&E sections from the medial-longitudinal plane of a male zebrafish reveals the main components of the digestive tract. Scale bar, 500 *μ*m. Click here for file

Additional file 2 **Overlap analysis of down-regulated genes in adjacent segments**. The number and percentage of overlapping genes are indicated within and below the intersection respectively. Click here for file

Additional file 3 **Expression of fabp2 gene in adult zebrafish intestine**. (A) Isolation of an intestine from a *Tg(fabp2:RFP) *fish. (B) Expression of *fabp2 *in a *Tg(fabp2:RFP) *fish as indicated by the RFP reporter. Circle, the junction where expression of *fabp2:rfp *transgene disappears. (C) In situ hybridization detection of endogenous fabp2 expression in adult zebrafish intestine from segment S1~S7, respectively. High expression level is observed in segments S1-S4, but it is turn off nearby the second natural turn of the intestine (circle). Beyond this region, the expression level becomes undetectable. Click here for file

Additional file 4 **Statistically enriched GO categories base on the GO Tree Machine program (p value < 0.01)**. Click here for file
