# Supplementary material for: Morphological and molecular evidence for functional organization along the rostrocaudal axis of the adult zebrafish intestine
Source: BMC Genomics. 2010 Jun 22;11:392. doi: 10.1186/1471-2164-11-392 (PMC2996925; doi:10.1186/1471-2164-11-392)

**Additional file 1**

**Anatomy of adult zebrafish showing the digestive tract.** A composite of H&E sections from the medial-longitudinal plane of a male zebrafish reveals the main components of the digestive tract. Scale bar, 500$\mu m$.


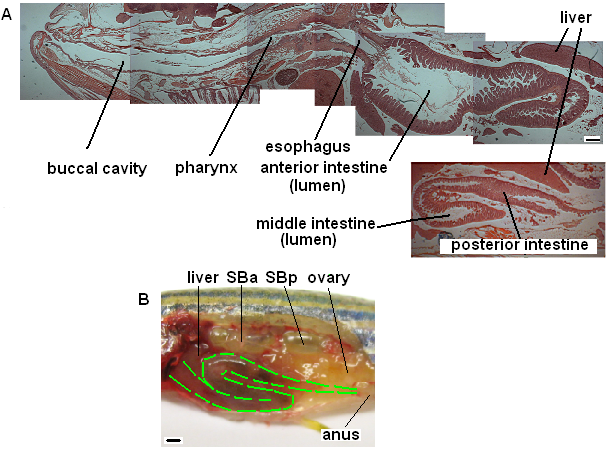

Supplement: Additional file 1 — Anatomy of adult zebrafish showing the digestive tract. A composite of H&E sections from the medial-longitudinal plane of a male zebrafish reveals the main components of the digestive tract. Scale bar, 500 μm. [file 1471-2164-11-392-S1.DOC]
