# Supplementary material for: Morphological and molecular evidence for functional organization along the rostrocaudal axis of the adult zebrafish intestine
Source: BMC Genomics. 2010 Jun 22;11:392. doi: 10.1186/1471-2164-11-392 (PMC2996925; doi:10.1186/1471-2164-11-392)

**Additional file 2**

**Overlap analysis of down-regulated genes in adjacent segments.** The number and percentage of overlapping genes are indicated within and below the intersection respectively.


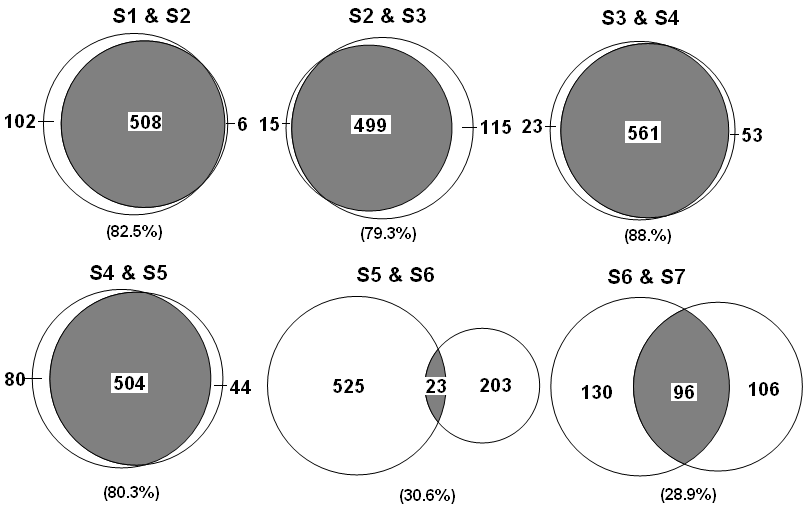

Supplement: Additional file 2 — Overlap analysis of down-regulated genes in adjacent segments. The number and percentage of overlapping genes are indicated within and below the intersection respectively. [file 1471-2164-11-392-S2.DOC]
