# Supplementary material for: Morphological and molecular evidence for functional organization along the rostrocaudal axis of the adult zebrafish intestine
Source: BMC Genomics. 2010 Jun 22;11:392. doi: 10.1186/1471-2164-11-392 (PMC2996925; doi:10.1186/1471-2164-11-392)

**Additional file 3**

**Expression of *fabp2* gene in adult zebrafish intestine.** (A) Isolation of an intestine from a *Tg(fabp2:RFP)* fish. (B) Expression of *fabp2* in a *Tg(fabp2:RFP)* fish as indicated by the RFP reporter. Circle, the junction where expression of *fabp2:rfp* transgene disappears. (C) In situ hybridization detection of endogenous *fabp2* expression in adult zebrafish intestine from segment S1~S7, respectively. High expression level is observed in segments S1-S4, but it is turn off nearby the second natural turn of the intestine (circle). Beyond this region, the expression level becomes undetectable.


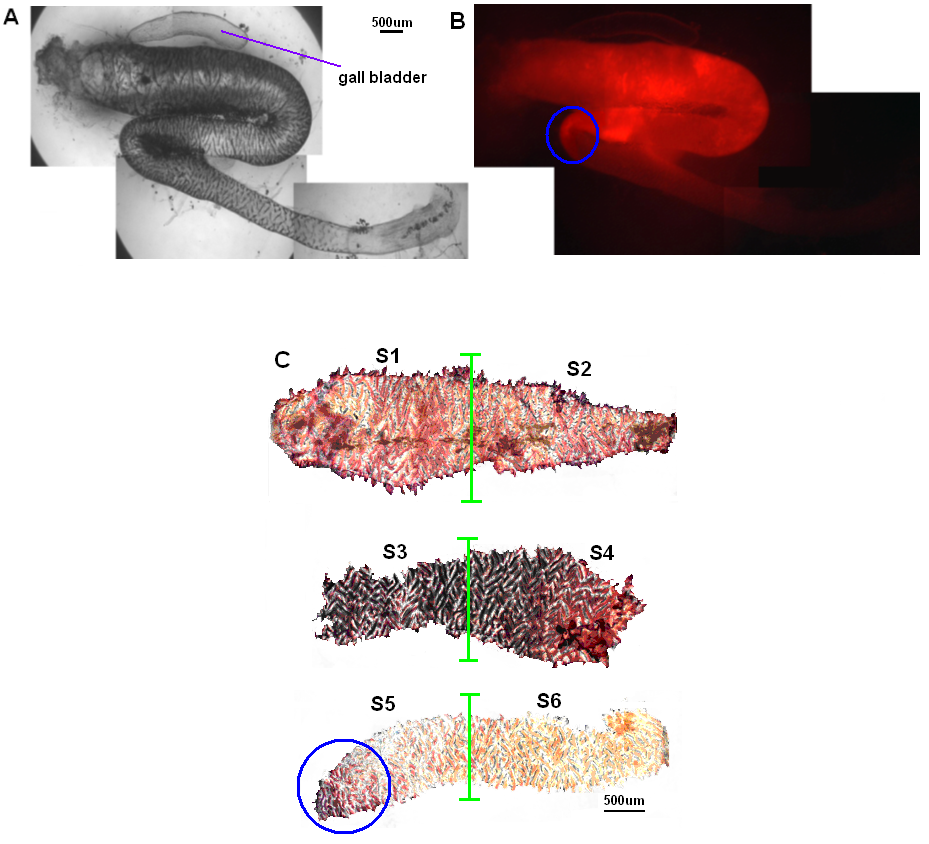

Supplement: Additional file 3 — Expression of fabp2 gene in adult zebrafish intestine. (A) Isolation of an intestine from a Tg(fabp2:RFP) fish. (B) Expression of fabp2 in a Tg(fabp2:RFP) fish as indicated by the RFP reporter. Circle, the junction where expression of fabp2:rfp transgene disappears. (C) In situ hybridization detection of endogenous fabp2 expression in adult zebrafish intestine from segment S1~S7, respectively. High expression level is observed in segments S1-S4, but it is turn off nearby the second natural turn of the intestine (circle). Beyond this region, the expression level becomes undetectable. [file 1471-2164-11-392-S3.DOC]
