# Supplementary material for: Morphological and molecular evidence for functional organization along the rostrocaudal axis of the adult zebrafish intestine
Source: BMC Genomics. 2010 Jun 22;11:392. doi: 10.1186/1471-2164-11-392 (PMC2996925; doi:10.1186/1471-2164-11-392)
Supplement: Additional file 4 — Statistically enriched GO categories base on the GO Tree Machine program (p value < 0.01). [file 1471-2164-11-392-S4.DOC]

**Additional file 4**

Statistically enriched GO categories base on the GO Tree Machine program (p value < 0.01)

| **Segment S1-S5** | | | | |
| --- | --- | --- | --- | --- |
| **Functional categories** | **Observed** | **Expected** | **Ratio (O/E)** | **P-value** |
| **In biologicial process** |  |  |  |  |
| vitamin biosynthesis | 3 | 0.08 | 37.5 | 1.55E-05 |
| water-soluble vitamin biosynthesis | 3 | 0.08 | 37.5 | 1.55E-05 |
| alcohol metabolism | 7 | 1.28 | 5.47 | 2.42E-04 |
| gluconeogenesis | 3 | 0.15 | 20 | 2.92E-04 |
| alcohol biosynthesis | 3 | 0.18 | 16.67 | 5.02E-04 |
| hexose biosynthesis | 3 | 0.18 | 16.67 | 5.02E-04 |
| monosaccharide biosynthesis | 3 | 0.18 | 16.67 | 5.02E-04 |
| pyruvate metabolism | 3 | 0.18 | 16.67 | 5.02E-04 |
| heme biosynthesis | 6 | 1.08 | 5.56 | 6.26E-04 |
| heme metabolism | 6 | 1.08 | 5.56 | 6.26E-04 |
| monosaccharide metabolism | 6 | 1.08 | 5.56 | 6.26E-04 |
| pigment biosynthesis | 6 | 1.08 | 5.56 | 6.26E-04 |
| pigment metabolism | 6 | 1.08 | 5.56 | 6.26E-04 |
| pyridine nucleotide biosynthesis | 2 | 0.05 | 40 | 6.31E-04 |
| vascular endothelial growth factor receptor signaling pathway | 2 | 0.05 | 40 | 6.31E-04 |
| heterocycle metabolism | 7 | 1.51 | 4.64 | 6.70E-04 |
| iron ion transport | 7 | 1.51 | 4.64 | 6.70E-04 |
| iron ion homeostasis | 7 | 1.53 | 4.58 | 7.41E-04 |
| transition metal ion homeostasis | 7 | 1.53 | 4.58 | 7.41E-04 |
| water-soluble vitamin metabolism | 3 | 0.2 | 15 | 7.88E-04 |
| secondary metabolism | 6 | 1.13 | 5.31 | 8.03E-04 |
| di-\, tri-valent inorganic cation homeostasis | 7 | 1.58 | 4.43 | 9.02E-04 |
| metal ion homeostasis | 7 | 1.58 | 4.43 | 9.02E-04 |
| porphyrin biosynthesis | 6 | 1.16 | 5.17 | 9.04E-04 |
| porphyrin metabolism | 6 | 1.16 | 5.17 | 9.04E-04 |
| generation of precursor metabolites and energy | 12 | 4.37 | 2.75 | 1.23E-03 |
| carboxylic acid metabolism | 8 | 2.16 | 3.7 | 1.28E-03 |
| organic acid metabolism | 8 | 2.16 | 3.7 | 1.28E-03 |
| cofactor metabolism | 9 | 2.69 | 3.35 | 1.31E-03 |
| cation homeostasis | 7 | 1.71 | 4.09 | 1.43E-03 |
| cell ion homeostasis | 7 | 1.73 | 4.05 | 1.55E-03 |
| cofactor biosynthesis | 8 | 2.24 | 3.57 | 1.60E-03 |
| ion homeostasis | 7 | 1.78 | 3.93 | 1.84E-03 |
| glucose metabolism | 5 | 0.9 | 5.56 | 1.85E-03 |
| cell homeostasis | 7 | 2.01 | 3.48 | 3.65E-03 |
| vitamin metabolism | 3 | 0.33 | 9.09 | 3.67E-03 |
| hexose metabolism | 5 | 1.08 | 4.63 | 4.11E-03 |
| homeostasis | 7 | 2.06 | 3.4 | 4.20E-03 |
| main pathways of carbohydrate metabolism | 5 | 1.16 | 4.31 | 5.51E-03 |
| carbohydrate biosynthesis | 3 | 0.38 | 7.89 | 5.63E-03 |
| energy derivation by oxidation of organic compounds | 5 | 1.18 | 4.24 | 6.04E-03 |
| **In molecular function** |  |  |  |  |
| fatty acid binding | 4 | 0.15 | 26.67 | 4.79E-06 |
| iron ion binding | 9 | 2.19 | 4.11 | 2.92E-04 |
| nicotinate phosphoribosyltransferase activity | 2 | 0.02 | 100 | 3.03E-04 |
| lipid binding | 6 | 1.12 | 5.36 | 7.67E-04 |
| catalytic activity | 46 | 32.34 | 1.42 | 3.20E-03 |
| acyl-CoA binding | 2 | 0.1 | 20 | 3.41E-03 |
| tetrapyrrole binding | 5 | 1.05 | 4.76 | 3.59E-03 |
| heme binding | 5 | 1.05 | 4.76 | 3.59E-03 |
| ligand-dependent nuclear receptor activity | 4 | 0.78 | 5.13 | 7.06E-03 |
| steroid hormone receptor activity | 4 | 0.78 | 5.13 | 7.06E-03 |
| cytoskeletal protein binding | 4 | 0.8 | 5 | 7.88E-03 |
| hydrolase activity\, acting on carbon-nitrogen (but not peptide) bonds\, in linear amides | 2 | 0.15 | 13.33 | 8.26E-03 |
| **In cellular component** |  |  |  |  |
| heterotrimeric G-protein  complex | 2 | 0.15 | 13.33 | 8.83E-03 |
| plasma membrane part | 6 | 1.81 | 3.31 | 8.98E-03 |

Segment S6

| **In biologicial process** |  |  |  |  |
| --- | --- | --- | --- | --- |
| one-carbon compound metabolism | 5 | 0.99 | 5.05 | 2.48E-03 |
| **In molecular function** |  |  |  |  |
| oxidoreductase activity\, acting on the aldehyde or oxo group of donors\, NAD or NADP as acceptor | 3 | 0.23 | 13.04 | 8.81E-04 |
| catalase activity | 2 | 0.05 | 40.00 | 1.11E-03 |
| oxidoreductase activity\, acting on the aldehyde or oxo group of donors | 3 | 0.37 | 8.11 | 4.45E-03 |
| **In cellular component** |  |  |  |  |
| contractile fiber | 2 | 0.04 | 50.00 | 9.09E-04 |
| contractile fiber part | 2 | 0.04 | 50.00 | 9.09E-04 |

Segment S7

| **In biologicial process** |  |  |  |  |
| --- | --- | --- | --- | --- |
| pyridine nucleotide biosynthesis | 2 | 0.08 | 25 | 1.78E-03 |
| intracellular signaling cascade | 16 | 7.6 | 2.11 | 3.44E-03 |
| vitamin biosynthesis | 2 | 0.13 | 15.38 | 5.11E-03 |
| water-soluble vitamin biosynthesis | 2 | 0.13 | 15.38 | 5.11E-03 |
| neuropeptide signaling pathway | 3 | 0.42 | 7.14 | 7.01E-03 |
| membrane organization and biogenesis | 2 | 0.17 | 11.76 | 9.94E-03 |
| **In molecular function** |  |  |  |  |
| transferase activity\, transferring pentosyl groups | 3 | 0.44 | 6.82 | 8.38E-03 |
| nicotinate phosphoribosyltransferase activity | 2 | 0.04 | 50.00 | 8.54E-04 |
| **In cellular component** |  |  |  |  |
| nil | - | - | - | - |
